# Alcohol consumption among university students in Ireland and the United Kingdom from 2002 to 2014: a systematic review

**DOI:** 10.1186/s12889-016-2843-1

**Published:** 2016-02-19

**Authors:** Martin P. Davoren, Jakob Demant, Frances Shiely, Ivan J. Perry

**Affiliations:** Department of Epidemiology and Public Health, University College Cork, 4th Floor Western Gateway Building, Western Road, Cork, Ireland; Department of Sociology, University of Copenhagen, Copenhagen, Denmark

**Keywords:** Alcohol, Prevalence, Review, Students

## Abstract

**Background:**

Alcohol is a leading cause of global suffering. Europe reports the uppermost volume of alcohol consumption in the world, with Ireland and the United Kingdom reporting the highest levels of binge drinking and drunkenness. Levels of consumption are elevated among university students. Thus, this literature review aims to summarise the current research on alcohol consumption among university students in the Republic of Ireland and the United Kingdom.

**Methods:**

MEDLINE, CINAHL, EMBASE and PsychInfo were systematically searched for literature from January 2002 until December 2014. Each database was searched using the following search pillars: alcohol, university student, Ireland or the United Kingdom and prevalence studies.

**Results:**

Two thousand one hundred twenty eight articles were retrieved from electronic database searching. These were title searched for relevance. 113 full texts were retrieved and assessed for eligibility. Of these, 29 articles were deemed to meet inclusion criteria for the review. Almost two thirds of students reported a hazardous alcohol consumption score on the AUDIT scale. Over 20 % reported alcohol problems over their lifetime using CAGE while over 20 % exceed sensible limits each week. Noteworthy is the narrowing of the gender gap throughout the past decade.

**Conclusion:**

This is the first review to investigate consumption patterns of university students in Ireland and the United Kingdom. A range of sampling strategies and screening tools are employed in alcohol research which preclude comparability. The current review provides an overview of consumption patterns to guide policy development.

## Background

Alcohol consumption is of considerable public health concern and a leading cause of global suffering [1]. Of particular concern are the health issues and social effects associated with its use [2–5]. Patterns of alcohol consumption range between continents and countries. Recent figures from the World Health Organisation (WHO) demonstrate that the European Region (E.U.) is the heaviest drinking region in the world [6]. Consumption levels peak in both the Nordic countries and the British Isles including the United Kingdom and Ireland [7, 8].

Elevated levels of alcohol consumption among young adults aged 18–29, of which university students represent a unique population, is of particular concern [9]. Research suggests that students today drink more, with increasing emphasis on binge drinking and drunkenness than among earlier generations [10–13]. Authors have previously hypothesised this as the ‘psychoactive revolution’ and by the 1990’s, a decade defined by a ‘new culture of intoxification’ had manifested which plateaued in 2001 [14]. Internationally, an extensive volume of research has been conducted to investigate the prevalence of hazardous alcohol consumption among students [15]. These studies range in methodological approaches and quality and have resulted in varying response rates. Also, unlike many other health behaviours which apply a standard approach to measurement, a variety of alcohol screening tools have been developed to categorise alcohol consumption levels. This impacts on the ability to compare and contrast when reviewing research in the area. A number of reviews of nations and continents have been undertaken.

Policy-makers and health system managers routinely legislate for complex issues such as alcohol consumption [16]. Systematic reviews are an integral feature of informing effective public health policy. Public policy makers “are less likely to be misled by results of a systematic review than a single investigation and can thus be more confident about the consequences a decision might produce” [16]. National strategies have highlighted the importance of tackling university student alcohol consumption when reducing population levels [17]. The Republic of Ireland and the United Kingdom both report high levels of harmful drinking among university students. Moreover, they provide state-funded universities which are independently run. These differ from Nordic countries which provide free undergraduate degrees to students. Thus, this literature review aims to summarise all available information on the prevalence of alcohol consumption among university students in the Republic of Ireland and the United Kingdom from 2002 to 2014.

## Methods

### Eligibility criteria

Following a scoping exercise, inclusion criteria for this review were as follows: 1) Cross-sectional studies which reported a prevalence of alcohol consumption, 2) Studies conducted within a university/college student population, 3) Studies conducted at universities or colleges in the United Kingdom or the Republic of Ireland and 4) studies published between January 1^st^ 2002 and December 31^st^ 2014. Any research article which did not correspond to each of these criteria was excluded.

### Information sources and search strategy

**Fig. 1:**
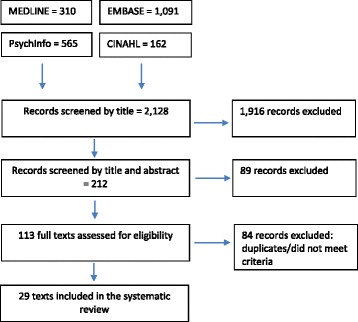
Flowchart of studies included in the review

MEDLINE, EMBASE, CINAHL and PsychInfo were systematically searched for literature from January 2002 until December 2014. For each database, searching was conducted using a combination of the following search terms: alcohol*, alcohol drinking, alcoholism, alcohol behaviour, university student*, College student*, Ireland, United Kingdom, Britain, prevalence, cross-sectional and questionnaire*. Search terms were combined using the Boolean logic of AND or OR operators. Completed searches were title searched for relevant articles by one reviewer (MPD). Irrelevant articles were excluded at this stage. All articles which referred to the research question were downloaded. Table and abstracts were analysed to investigate suitability (MPD and FS) and relevant articles were fully reviewed (MPD and FS). A flow diagram of this is displayed in Fig. 1. References for all included articles were managed in Endnote, a reference package, to keep track of paper selection.

### Screening tools

Data extraction highlighted a number of screening tools available to university students.

The Alcohol Use Disorders Identification Test (AUDIT) was developed by the World Health Organisation to identify excessive drinkers. This screening tool identifies hazardous patterns of alcohol consumption. The AUDIT-C takes the first three questions of the AUDIT questionnaire. These questions focus on the frequency of consumption, the number of units consumed and the number of binge drinking occasions. The guidelines are provided on safe alcohol consumption. Separate guidelines are provided for men and women with lower low-risk thresholds for women than those for men reflecting their increased vulnerability to alcohol related harm [18].

The CAGE questionnaire is favoured in the primary care setting due to the fact that it is short and easy to remember. It asks four questions; *Have you ever felt you should cut down on your drinking? Have people annoyed you by criticising your drinking? Have you ever had a drink first thing in the morning to steady your nerves or to get rid of a hangover?* The CAGE can identify alcohol problems over the lifetime. Two positive responses are considered a positive test and indicate further assessment is warranted.

The remaining studies, eligible for inclusion in this review, employed the screening tool FAST or the number of units consumed as a measure of risky drinking behaviour. Authors used binge drinking which is defined as six or more units in one drinking session. Other studies noted the number of weekly units exceeding nationally recommended weekly limits.

### Data items and quality assessment

Data extraction forms were utilised in the current research. Data was extracted on sociodemographic, methodological and alcohol information by one author (MPD). This was reviewed by a co-author (FS) and any disagreements discussed with a third co-author (IJP). Data was extracted under the following headings:

1. Publication details: author(s) and year of study
2. Design: study design, sample size, response rate, name of screening tool(s)
3. Study participant details: age, sex, graduate course, period of study
4. Prevalence of hazardous or harmful alcohol consumption, binge drinking and exceeding weekly limits

Table 1 displays the results of the quality assessment Quality assessment was undertaken using the quality assessment tool outlined by Loney et al [19] to appraise prevalence studies. The tool investigates three main areas: 1) Are the study methods valid, 2) What is the interpretation of the results? and 3) What is the applicability of the results? Each criteria was given a point and each study marked out of a total of eight.

**Table 1:** Quality assessment of included studies investigating drinking patterns of Irish and UK undergraduate students (n = 29)

Study	Random sample?	Unbiased sampling frame	Sample size?	Measures?	Unbiased assessors?	Response rate?	Confidence intervals?	Subject description?	Total
Black & Monrouxe, 2014 [39]	-	✓	-	✓	✓	-	-	✓	4/8
El Ansari et al, 2014 [29]	✓	✓	✓	✓	✓	✓	✓	✓	8/8
John & Alwyn, 2014 [41]	-	-	-	✓	✓	-	-	✓	3/8
O’Brien et al, 2014 [24]	-	-	✓	✓	-	✓	✓	✓	5/8
El ansari et al, 2013 [28]	✓	✓	✓	✓	✓	✓	✓	✓	8/8
Partington et al, 2013 [25]	-	✓	-	✓	✓	-	-	✓	4/8
De Visser & McDonnell, 2012 [40]	✓	✓	-	✓	✓	-	-	✓	5/8
Gardner et al, 2012 [35]	-	-	-	✓	✓	-	-	-	2/8
Gunby et al, 2012 [22]	✓	-	-	✓	✓	-	-	✓	4/8
Sebena et al, 2012 [31]	-	-	✓	✓	✓	✓	✓	✓	6/8
Craigs et al, 2011 [45]	✓	✓	-	✓	✓	✓	-	✓	6/8
El Ansari et al, 2011 [30]	✓	✓	✓	✓	✓	✓	✓	✓	8/8
Heather et al, 2011 [23]	✓	✓	-	✓	✓	-	-	✓	5/8
Beenstock et al, 2010 [20]	✓	-	-	✓	✓	✓	✓	✓	6/8
Cahill & Byrne, 2010 [32]	✓	✓	-	✓	✓	✓	-	-	5/8
Dodd et al, 2010 [33]	-	-	-	✓	✓	-	✓	✓	4/8
El Ansari & Stock, 2010 [34]	✓	✓	✓	✓	✓	✓	✓	✓	8/8
Woolfson & Maguire, 2010	✓	-	-	✓	✓	-	-	-	3/8
Underwood et al, 2009 [47]	-	-	-	✓	✓	-	-	-	2/8
O’Connor et al, 2008 [48]	✓	-	-	✓	✓	✓	-	-	4/8
Gill et al, 2007 [36]	✓	-	-	✓	✓	-	-	✓	4/8
Norman et al, 2007 [37]	-	-	-	✓	✓	-	-	-	2/8
Barber & Fairclough, 2006 [60]	-	-	-	✓	✓	✓	-	✓	4/8
Boland et al, 2006 [27]	-	-	-	✓	✓	✓	-	✓	4/8
Faulkner et al, 2006 [21]	-	-	-	✓	-	-	-	-	1/8
Watson et al, 2006 [43]	-	-	-	✓	✓	✓	-	✓	4/8
McMillan & Conner, 2003 [42]	-	-	-	✓	✓	-	-	-	2/8
Snow et al, 2003 [26]	-	-	-	✓	✓	-	-	-	2/8
Newbury-Birch et al, 2002 [46]	-	-	-	✓	✓	✓	-	-	3/8

**Table 2:** Summary of research studies investigating drinking patterns of Irish and UK undergraduate students

Study	Year	Design	Graduate course	Period of course	Sample size/Response rate	Age	Sex	Screening tool	Prevalence
Black & Monrouxe, 2014 [39]	-	Method was dependent on each institution (e-mail, online notice board, and social media). Students informed others of the questionnaire (i.e. snowball sampling).	Medicine	All medical years included	216 individuals responded/-	17-25	Men and Women	Weekly alcohol consumption. 15 units or more/week	21.6 %/weekly
El Ansari et al, 2014 [29]	2007–2008	Self-administered questionnaire were provided to students during the last 10 minutes of class time.	All degree programmes	First-third year	3,220/80 %	Mean range: 22.2–31.6	Men and Women	Frequency of HED^a^/past week CAGE	59.2 % - HED^a^ 22.4 % - problem drinking 8.8 % - alcohol dependence.
John & Alwyn, 2014 [41]	-	Questionnaires were distributed and returned at formal teaching sessions	-	Undergraduate, first year student	374 First year students	18–22	Men and Women	FAST/typical week	85 % - Binge drinking occasions (8 units or more in one sitting)/typical week
O’Brien et al, 2014 [24]	September 2010–February 2012	Individuals were sampled at venues leased or owned by the university at events noted from webpage listings or competition schedules.	-	-	2,048/83 %	Mean = 19.97	Men and women	AUDIT	84 % - hazardous drinkers
El ansari et al, 2013 [28]	2007–2008	A self-completed questionnaire was distributed during the last 10 minute of lectures.	All degree programmes	First-third year	3,706 students/80 %	24.9 years	Men and women	CAGE	67.2 % - HED 22.4 % - Problem drinking 8.8 % - alcohol dependence.
Partington et al, 2013 [25]	March 08-March 09	Cross-sectional study – questionnaire booklet distributed either at the start or end of lectures	Science or arts based programmes	First-third year	770/-	Median – 22	Men and women	AUDIT	60.6 % - hazardous pattern of drinking: Hazardous – 40.1 % Harmful – 10.9 % Probable dependence – 9.6 %
De Visser & McDonnell, 2012 [40]	-	An online questionnaire investigating various lifestyle and health-related beliefs and behaviours was sent to students inviting them to complete	Not specified	Not specified	731/-	18-25 years	Men and women	Units of alcohol consumed/past week	47.5 % of women and 51.3 % of men noted exceeding their weekly unit guidelines/past week.
Gardner et al, 2012 [35]	-	The questionnaire was posted online and sent to students for completion. Students were advised to forward the e-mail to other students.	Undergraduate students	All undergraduate years	167/-	18-35 years. Mean – 21.95	Men and women	Binge drinking/past week	56.2 % reported at least one binge drinking session/past week
Gunby et al, 2012 [22]	2008 –2009	Students received an e-mail inviting them to participate in the research. In addition, posters inviting students to participate were also displayed on campus.	Undergraduate and postgraduate students	All years	1,110/-	18-24 years	Men and women	AUDIT	71.2 % - hazardous alcohol consumption.
Sebena et al, 2012 [31]	2007	Students received the questionnaire to complete during class time.	All first year courses	First year	2,529/74 %	Not specified	Men and women	CAGE	Problem drinking: 22.1 % (CAGE score of 2 or more)
Craigs et al, 2011 [45]	2007/08	Participants were asked to complete a questionnaire electronically	Undergraduate	All academic years included	119/73 %	Mean age – 22.87 years	Men and women	Units of alcohol consumed/week	32 % - hazardous drinkers/week
El Ansari et al, 2011 [30]	2007–2008	A self-completed questionnaire was distributed during the last 10 minute of lectures.	Modules included: Social sciences, sport, sport development and exercise, health science	First-third year	3,706/80 %	24.9 years	Men and women	CAGE	23.1 % - problem drinking
Heather et al, 2011 [23]	2008–2009	Cross-sectional study – questionnaire booklet distributed either at the start or end of lectures.	Students registered to science based or arts based programmes. The five most popular subject areas were targeted.	First, second and third year	770/-	Mean – 22.3	Men and women	AUDIT	Positive AUDIT – 60.6 % Hazardous – 40.1 % Harmful – 10.9 % Probable dependence – 9.6 %
Beenstock et al, 2010 [20]	2008	Questionnaires were distributed at select lecture theatres. Following this, questionnaires were posted on the students online learning system, BlackBoard.	Deans of undergraduate studies in the faculties of Humanities, Social Science and Agriculture & Engineering were asked to participate.	Questionnaires were distributed across all years of study.	458/67 %	20 years - median	Men and women	AUDIT	82 % positive AUDIT. 39 % hazardous drinkers, 22 % harmful drinkers and 21 % may be alcohol dependent.
Cahill & Byrne, 2010 [32]	2008	Students who attended the Student Health Department over two days in November 2008 were asked to complete an anonymous survey on alcohol and drug use.	Students from any faculty	Students from any year	181/91.4 %	Over half <21	Men and women	Units of alcohol consumed/week	83.4 % - binge drinking in the previous 12 months. 44.8 % binge drink once weekly.
Dodd et al, 2010 [33]	-	Convenience sampling was utilised with the selection recruited where time for data collection was given.	-	First -fourth year students from one UK university	410/16 %	22.8 years – mean age	Men and women	Binge drinking/past week	Binge drinking was reported by 65.4 % of men (5 or more units) and 52.6 % of women (4 or more units)/past week
El Ansari & Stock, 2010 [34]	2008–2009	Two cross-sectional surveys were undertaken simultaneously using a universal sampling strategy (all students invited). Data was collected at selected modules at the end of teaching sessions.	Undergraduate students	All academic years included	380 students: 195 male and 185 female	22.86 years – Mean	Men and women	Binge drinking frequency/past month	11.5 % - binge drinking 10 times or more in the last month; 15.8 % - binge drinking (5 or more drinks in a row) 6-9 times in past month. 26.5 % reported binge drinking 3-5 times in the past month. 12.9 % reported it 1 and 2 times. 20.4 % reported not binge drinking/previous month.
Woolfson & Maguire, 2010	2007	The university portal was used for student to opt-in and complete the questionnaire online over a 4 week period.	-	-	62/96.9 %	21 years – Mean age	Men and women	Binge Drinking (units)/monthly	82.3 % reported partaking in binge drinking sessions (^b^)in the four week period of follow up.
Underwood et al, 2009 [47]	2008	2^nd^, 3^rd^ and 4^th^ year students completed the questionnaire before scheduled lectures. Absentees from class, 1^st^ and 5^th^ year students were contacted via internal mail.	Dental undergraduate students	First to fifth year dental students	384/-	-	Men and women	Hazardous from units consumed (50 or more for men, 36 or more for women)/week.	In 2008, 1.7 % of males and 2.6 % of females reported hazardous alcohol consumption last week. 0 % of men and 2.7 % of women report hazardous alcohol consumption on an average week.
O’Connor et al, 2008 [48]	2003	Questionnaires were distributed around the library and collected approximately 30 minutes later. The method was designed to capture a representative sample of the UCC student population.	Undergraduate and postgraduate students	All years included.	115/100 % response rate	Undergraduates – 20.2 years Graduates – 44.2 years	Men and women	Units of alcohol consumed ^c^/week	One third (32.1 %) of the UCC undergraduates were in the risky drinking category/week.
Gill et al, 2007 [36]	-	Second year students were at informed about the study at the beginning of class and were asked to complete the questionnaire a week later in an adjacent classroom.	Undergraduate students	Second year undergraduate students registered to one university	95	20.1 years (18.1–25.3) – mean age	Female only	Units of alcohol consumed^c^/past week	70 % of individuals reported binge drinking^c^ one day within the previous 7 days
Norman et al, 2007 [37]		Questionnaires were completed by a sample of undergraduate students at two time-points. Theory of planned behaviour and binge drinking questions were included.	Undergraduate students	Not specified	94/84 %	20.1 – Mean age	Men and women	Binge drinking^b^/past week	73.4 % - binge drinking/past week (T1) 62.0 % - binge drinking/past week (T2)
Barber & Fairclough, 2006 [60]	2001	Questionnaires were distributed to all dental students and selected law students during lectures and returned via an enclosed response box placed in the lobby area of each faculty.	Dental and Law undergraduate students	Each year	All dental students were selected. 180 law students/-Dental:83 %, Law: 71 %	Mean age: Dental students – 21.4 years, Law students – 20.4 years	Men and women	Alcohol use categorised to sensible^c^	30 % of dental students and 40 % of Law students reported exceeding sensible levels of alcohol consumption^c^/week
Boland et al, 2006 [27]	2002	A researcher distributed the surveys to students in class, outlined the aims and objectives of the research and collected the completed surveys before departing.	Medical students	All undergraduate years of medicine	537/63 %	19–22	Men and women	CAGE	2002 – 52.5 % of medical students reported a positive CAGE score
Faulkner et al, 2006 [21]	-	Students living in halls of residence in a campus university in South Wales were eligible for inclusion. Questionnaires were distributed to as many halls as could be accessed and collected two days later.	-	-	282/47 %	Mean age – 20.2 years	Men and women	AUDIT	AUDIT scores in excess of 8 were reported by 85 % of males and 73 % of females.
Watson et al, 2006 [43]	-	Questionnaires were distributed at the end of lectures.	Undergraduate nursing students	First year (pre-registration) nursing and midwifery students	186/93 %	The majority of students were aged 17-30	Men and women	Units of alcohol consumed ^de^/week	86.5 % reported having drunk alcohol on at least one occasion in the previous week. 74 % of students report drinking at levels above low risk drinking + ./week 54.7 % of students reported binge drinking^e^/week
McMillan & Conner, 2003 [42]	-	Respondents were recruited at lectures.	Undergraduate students	All years were eligible for inclusion in this study	A response rate of 62 % was noted.	17-54 years	Men and women	Units of alcohol consumed ^c^/week	65.2 % of men and 40.7 % of women report exceeding ‘sensible’ levels/week 19.6 % of men and 1.2 % of women reported hazardous drinking/week
Snow et al, 2003 [26]	-	Questionnaires were completed by a convenience sample of individuals who expressed interest in participating in focus groups.	Psychology, Law and Business	Undergraduate first year students	300 questionnaires distributed 187 students - 62 % response rate	Mean age – 20.2 years	Men and women	AUDIT	69.2 % of males and 62.8 % of females had a hazardous AUDIT score.
Newbury-Birch et al, 2002 [46]	1995 and 1998	Second year students followed up in final year. Questionnaires were distributed in class or group meetings for completion. Attendance was monitored and absent students received the questionnaire in the post.	Undergraduate medical and dental students	Second and final year	427/71-80 %	-	Men and women	Units of alcohol consumed ^c^/week	37.5 % of men and 9.7 % of women as second years were hazardous drinkers/week. As final years it was reported by 7.4 % of men and no women/week.

^a^HED: consuming 5 or more alcohol drinks in one sitting over the last two weeks

^b^Binge drinking: five pints of beer, 10 shorts or glasses of wine in a single session for men, 3.5 pints of beer, seven shorts or glasses of wine for women

^c^Exceeding sensible limits notes the WHO weekly limits of 21 units or more for men and 14 units or more for women

^d^3-4 units/day but not exceeding 21 units/week for men. 2-3 units/day but not exceeding 14 units/week for women

^e^Over 8 units on one occasion for men, over 6 units on one occasion for women

**Table 3:** PRISMA checklist for systematic reviews

Section/topic	#	Checklist item	✓
TITLE			
Title	1	Identify the report as a systematic review, meta-analysis, or both.	✓
ABSTRACT			
Structured summary	2	Provide a structured summary including, as applicable: background; objectives; data sources; study eligibility criteria, participants, and interventions; study appraisal and synthesis methods; results; limitations; conclusions and implications of key findings; systematic review registration number.	✓
INTRODUCTION			
Rationale	3	Describe the rationale for the review in the context of what is already known.	✓
Objectives	4	Provide an explicit statement of questions being addressed with reference to participants, interventions, comparisons, outcomes, and study design (PICOS).	✓
METHODS			
Protocol and registration	5	Indicate if a review protocol exists, if and where it can be accessed (e.g., Web address), and, if available, provide registration information including registration number.	-
Eligibility criteria	6	Specify study characteristics (e.g., PICOS, length of follow-up) and report characteristics (e.g., years considered, language, publication status) used as criteria for eligibility, giving rationale.	✓
Information sources	7	Describe all information sources (e.g., databases with dates of coverage, contact with study authors to identify additional studies) in the search and date last searched.	✓
Search	8	Present full electronic search strategy for at least one database, including any limits used, such that it could be repeated.	✓
Study selection	9	State the process for selecting studies (i.e., screening, eligibility, included in systematic review, and, if applicable, included in the meta-analysis).	✓
Data collection process	10	Describe method of data extraction from reports (e.g., piloted forms, independently, in duplicate) and any processes for obtaining and confirming data from investigators.	✓
Data items	11	List and define all variables for which data were sought (e.g., PICOS, funding sources) and any assumptions and simplifications made.	✓
Risk of bias in individual studies	12	Describe methods used for assessing risk of bias of individual studies (including specification of whether this was done at the study or outcome level), and how this information is to be used in any data synthesis.	✓
Summary measures	13	State the principal summary measures (e.g., risk ratio, difference in means).	✓
Synthesis of results	14	Describe the methods of handling data and combining results of studies, if done, including measures of consistency (e.g., I^2^) for each meta-analysis.	✓
Risk of bias across studies	15	Specify any assessment of risk of bias that may affect the cumulative evidence (e.g., publication bias, selective reporting within studies).	-
Additional analyses	16	Describe methods of additional analyses (e.g., sensitivity or subgroup analyses, meta-regression), if done, indicating which were pre-specified.	-
RESULTS			
Study selection	17	Give numbers of studies screened, assessed for eligibility, and included in the review, with reasons for exclusions at each stage, ideally with a flow diagram.	✓
Study characteristics	18	For each study, present characteristics for which data were extracted (e.g., study size, PICOS, follow-up period) and provide the citations.	✓
Risk of bias within studies	19	Present data on risk of bias of each study and, if available, any outcome level assessment (see item 12).	✓
Results of individual studies	20	For all outcomes considered (benefits or harms), present, for each study: (a) simple summary data for each intervention group (b) effect estimates and confidence intervals, ideally with a forest plot.	✓
Synthesis of results	21	Present results of each meta-analysis done, including confidence intervals and measures of consistency.	✓
Risk of bias across studies	22	Present results of any assessment of risk of bias across studies (see Item 15).	-
Additional analysis	23	Give results of additional analyses, if done (e.g., sensitivity or subgroup analyses, meta-regression [see Item 16]).	-
DISCUSSION			
Summary of evidence	24	Summarize the main findings including the strength of evidence for each main outcome; consider their relevance to key groups (e.g., healthcare providers, users, and policy makers).	✓
Limitations	25	Discuss limitations at study and outcome level (e.g., risk of bias), and at review-level (e.g., incomplete retrieval of identified research, reporting bias).	✓
Conclusions	26	Provide a general interpretation of the results in the context of other evidence, and implications for future research.	✓
FUNDING			
Funding	27	Describe sources of funding for the systematic review and other support (e.g., supply of data); role of funders for the systematic review.	-

### Data synthesis

Relevant data extracted from eligible studies are presented in Table 2. Due to the number of different screening tools and variety of sampling strategies, a narrative review was conducted. This yielded a summary of the prevalence of alcohol consumption according to screening tool and further subdivided by gender when possible.

## Results

### Study characteristics

Figure 1 displays the results of the search strategy. Of the included studies, seven employed the AUDIT scale [20–26]. This is based on the frequency of consumption, the number of units consumed, the number of binge drinking occasions along with a range of second-hand effects of excessive alcohol use. A further five studies employed the CAGE questionnaire [27–31]. However, different cut-off scores were used across these studies. Seventeen studies questioned students on the units of alcohol they consumed. Of these seven describe binge drinking patterns [32–38], 6 describe exceeding sensible limits of weekly consumption [39–44], 3 describe hazardous drinking [45–47] and 1 risky drinking [48]. A proportion of studies reported consumption patterns by gender (n = 15).

In addition, a number of different sampling strategies were reported. These ranged in cluster size from students registered to a number of university campuses across the UK, to students in one university, to students in one faculty or department. Summaries of each study are displayed in Table 2.

### Summary of results

#### AUDIT

Among studies which employed the AUDIT scale, the proportion of students reporting hazardous alcohol consumption ranged from 62.8 % in 2003 to 84 % in 2014. In 2010, Beenstock reported results from a cross-sectional survey of university students at a university in Northern England. Using a university-wide sampling frame, 82 % reported an AUDIT score of eight or more, a rise in previous years. In 2011, Heather reported on hazardous alcohol consumption across seven universities in the United Kingdom. 60.6 % of the sample reported hazardous alcohol consumption. In 2012 Gunby reported results from a university in North West of England which found 71.2 % of students reported hazardous alcohol consumption. When sports students were questioned, O’Brien reported 84 % were hazardous alcohol consumers after delivering questionnaires to all sports venues in a 2 mile radius of ten universities across England.

The proportions of male and female hazardous consumption was reported in a number of articles. In 2003, Snow et al, reported a prevalence of 69.2 % among males and 62.8 % among females. However, this was a small sample of 187 students who had expressed interest in a focus group also being undertaken. Faulkner et al chose students living at halls of residence in a South Wales university. This study found 85 % of males and 73 % of females reporting a hazardous alcohol consumption score using the AUDIT scale in 2006. In 2010, Beenstock et al reported 89.1 % of men and 77.2 % of women having an AUDIT-C score of 8 or more. Reporting on multi-centre study, Heather noted that no significant difference in the HAC scores of men and women. A year later, Gunby noted a significant difference in the proportion of male (76.8 %) and female (69.4 %) hazardous alcohol consumers. However, O’Brien did not note any significant differences in male and female drinking patterns in their 2014 article [20–26].

#### CAGE

Among the five studies which employed the CAGE screening tool, the proportion of students reporting alcohol problems ranged from 22 to 76 %. In 2011, El Ansari reported findings that 23.1 % of students were problem drinkers from a study of seven participating universities. Similarly, Sebena reported 22.1 % of first year students registered to one English university reported problem drinking while El Ansari, 2013 noted 22.4 % of students across seven universities in the UK were problem drinkers.

In 2006, Boland et al found that 40.7 % of men and 27.8 % of women reported a positive CAGE score in 1990 which increased to 61.2 % of men and 46.6 % of women in 2002. A shift in drinking patterns was noted a decade later with females now reporting problem drinking (31 %) more than their male counterparts (21.2 %). One of the largest studies was conducted across seven universities in the United Kingdom. It contradicts this, noting 29.3 % of men report problem drinking compared to 20.4 % of women [27–31].

#### Units

The remaining 17 studies gave information on the number of units consumed by students. These studies describe students as heavy drinkers, hazardous drinkers, binge drinkers or drinkers who exceed sensible limits. For those papers which reported binge drinking, Gill et al noted that 70 % of students were binge drinking in the past week in 2007. In 2014, John et al noted that 85 % of students reported exceeding binge drinking limits on a typical week. A high of 83.4 % reporting binge drinking in the past 12 months was noted among Irish university students in 2010. Among those defined as hazardous drinkers per week, 37.5 % of men and 9.7 % of women were noted in 2002 which increased to 32 % of students per week by 2011. Papers which employed sensible limits of alcohol consumption highlight that levels of consumption for men in 2012 is similar to that in 2003. The latest study indicates that 21.6 % of students report consuming 15 units or more weekly [32–49].

## Discussion

The breadth of literature published on university student consumption highlights alcohol as the leading cause of concern among this sub-section of society [50, 51]. Although recent research has noted decreasing levels of alcohol consumption among young adults, hazardous alcohol consumption continues among university students in Ireland [15] and the United Kingdom [23]. This review highlights the high levels of alcohol consumption among university students and the narrowing proportions of risky drinking in male and female students. A generation of intoxication occurred in the 1990’s following a static period for alcohol consumption during the 70’s and 80’s. Since its peak in 2001, drunkenness and binge drinking have become commonplace among young adults [14]. However, it is difficult to conclude whether excessive consumption is due to a cultural shift in consumption patterns or as a direct result of alcohol marketing.

The current review highlights a range of issues that present themselves when synthesising results from alcohol research studies. Firstly, the representative nature of included studies ranged significantly. A variety of sampling procedures and populations in each research article was reported. Included articles ranged from medical students, a cross-section of students from a single university or a cross-section of a number of universities across the UK or Ireland. The most prominent issue with cross-sectional research is selection bias. This occurs as non-participation in surveys is rarely random [52]. Levin noted that “the sample frame used to select a sample and the response rate determine how well results can be generalised to the population as a whole“ [53]. Large scale cross-sectional studies are optimum as they sample the whole population. When the sampling frame is narrow, the concern of the researcher is that the sample will differ from the general population and results cannot be generalised, thereby reducing the external validity. When interpreting these results, the reader needs to consider systematic error (difference between the sample and the population) and particularly coverage error for studies which focused solely on medical or dental professions [54].

Secondly, in contrast to many other risk taking behaviours such as smoking or illicit drug use, alcohol can be considered both a protective (cardiovascular disease) and a risk factor (cancer) at low doses [55]. Much research has been conducted into devising screening tools for categorising harmful and non-harmful consumption [56–58]. A plethora of screening tools are now available and validated in both general and specific populations. The difficulty of having a broad variety of screening tools is that comparison is compromised when countries or institutions within countries employ different screening tools. The current review notes the use of AUDIT, CAGE and units. In addition, other screening tools such as FAST, RAPT and T-ACE have been developed and validated for use.

The current review supports our recent research indicating that patterns of alcohol consumption among male and female students are converging [15]. The past two decades has seen an increase in female alcohol consumption, the inauguration and continuation of ‘ladette culture’ and a focused effort on marketing alcohol directly at young female women [59]. The implications of this consumption is more serious given women’s innate biological susceptibility to the harms associated with alcohol consumption [2].

Tackling alcohol related harm among university students and the general population does not have a single solution but requires a suite of initiatives. Tax increases, minimum unit pricing, restricted access to retail alcohol and bans on alcohol advertising have been proven to effectively reduce the levels of hazardous drinking and alcohol related harm. Ireland and Scotland recently proposed the implementation of a minimum unit price for alcohol. Furthermore, the Irish government have committed to a suite of measures directed toward advertising, marketing and sponsorship being reviewed in three years, exposing their commitment to tackling this public health issue into the future.

### Strengths and weaknesses

The current review gives a broad overview of alcohol consumption patterns among university students since 2002. The PRISMA checklist was utilised to guide the review process (Table 3). The search strategy was conducted using a number of different search engines which yielded relevant literature from a wide range of medical and psycho-social disciplines. This provides synthesised information for policy-makers to draw upon [16].

This review also has a number of limitations. The interpretation of the findings of this review was restricted due to varying methods and tools used in calculating hazardous alcohol consumption. Recently, the ‘Cochrane Handbook for Systematic Reviews of Interventions’ notes that if studies are clinically diverse then a meta-analysis may be meaningless. A particularly important type of diversity is in the comparisons being made by the primary studies. The current review combines studies which have employed different sampling strategies, different methods of data collection and opposing screening tools. Furthermore, it was advised that conducting meta-analyses of studies that are at risk of bias may be seriously misleading. Thus a meta-analysis has not been conducted in the current review due to the level of heterogeneity observed. In addition, one unit of alcohol is measured differently in Ireland and the United Kingdom, the impact of which was not controlled for in this review. However, this review does highlight the prominent consumption patterns among university students across these similar university environments.

## Conclusion

Hazardous alcohol consumption continues to be the most prevalent public health issue encountered by university students. Despite increased efforts, levels of consumption among students have continued to increase throughout the past number of decades. These levels of consumption remain a primary concern to those attempting to improve student health and well-being. The current research provides public policy makers with an up-to-date summary of research to guide prevention efforts. As nations attempt to reduce alcohol related harm, a spotlight on the excessive consumption patterns among university students showcases the need for interventions to achieve national goals.

## Abbreviations

AUDIT: Alcohol Use Disorders Identification Test
EU: European Union
HAC: Hazardous Alcohol Consumption
UK: United Kingdom
WHO: World Health Organisation
